# Oral microbiota may affect osteoradionecrosis following radiotherapy for head and neck cancer

**DOI:** 10.1186/s12967-023-04219-y

**Published:** 2023-06-17

**Authors:** Zhengrui Li, Rao Fu, Xufeng Huang, Xutao Wen, Ling Zhang

**Affiliations:** 1 Department of Oral and Maxillofacial-Head and Neck Oncology, Shanghai Ninth People’s Hospital, Shanghai Jiao Tong University School of Medicine, Shanghai, China; 2 College of Stomatology, Shanghai Jiao Tong University, Shanghai, China; 3 National Center for Stomatology, Shanghai, China; 4 National Clinical Research Center for Oral Diseases, Shanghai, China; 5 Shanghai Key Laboratory of Stomatology, Shanghai, China; 6 Shanghai Research Institute of Stomatology, Shanghai, China; 7 Shanghai Center of Head and Neck Oncology Clinical and Translational Science, Shanghai, China; 8 Faculty of Dentistry, University of Debrecen, Debrecen, 4032 Hungary

**Keywords:** Microorganism, Osteoradionecrosis, 16S rRNA sequencing, Microbiota, Infectious disease

## Abstract

**Background:**

Osteoradionecrosis (ORN) is a serious complication of radiotherapy for head and neck cancer (HNC). However, its etiology and pathogenesis have not been completely elucidated. Recent studies suggest the involvement of the oral microbiota in the development of ORN. The aim of this study was to assess the correlation between oral microbiota and the extent of bone resorption in ORN patients.

**Materials and methods:**

Thirty patients who received high-dose radiotherapy for HNC were enrolled. Tissue specimens were collected from the unaffected and affected sides. The diversity, species differences and marker species of the oral microbial community were determined by 16 S rRNA sequencing and bioinformatics analysis.

**Results:**

The ORN group had greater microbial abundance and species diversity. The relative abundance of f_*Prevotellaceaeand*, f_*Fusobacteriaceae*, f_*Porphyromonadaceae*, f_*Actinomycetaceae*, f_*Staphylococcaceae*, g_*Prevotella*, g_*Staphylococcus*, s_*Endodontalis* and s_*Intermedia* were particular;y increased in ORN, suggesting a potential association between the oral microbiota and ORN. Furthermore, g_*Prevotella*, g_*Streptococcus*, s_*parvula and s_mucilaginosa* were identified as potential diagnostic and prognostic biomarkers of ORN. Association network analysis also suggested an overall imbalance in species diversity and ecological diversity in the oral microbiota of ORN patients. In addition, pathway analysis indicated that the dominant microbiota in ORN may disrupt bone regeneration by regulating specific metabolic pathways that increase osteoclastic activity.

**Conclusion:**

Radiation-induced ORN is associated with significant changes in the oral microbiota, and the latter may play a potential role in the etiopathology of post-radiation ORN. The exact mechanisms through which the oral microbiota influence osteogenesis and osteoclastogenesis remain to be elucidated.

## Introduction

Osteoradionecrosis (ORN) is one of the most serious complications of radiotherapy for head and neck cancers (HNC) [1], and its incidence rate ranges from 0.4–56% [2]. ORN manifests as local circulatory disorders, degeneration and necrosis of various cells in the bone and bone marrow, and impaired bone tissue repair [3]. Although the combination of surgery and radiotherapy is effective against HNC, radiation-induced ORN significantly worsens the quality of life for the patients. The exact etiology and pathogenesis of ORN remain unclear, although “radiation trauma and infection”, “bone damage” and “three low” (Blood vessel density, Bone cell activity and Bone tissue oxygen content decreased in jaw) have been implicated [4]. Radiation damages blood vessels and osteocytes, which lowers cellular activity and vascular density and induces local tissue hypoxia, resulting in bone destruction [5]. In addition, periosteal fibrosis and necrosis of osteoblasts and osteocytes lead to a substantial increase in the osteoclasts, resulting in bone resorption and increased risk of fractures [6]. Such vital bone loss can progress to infected osteoradionecrosis (IORN), which manifests as fever, pain, and the appearance of fistulas and inflammation in the surrounding mucosa or skin [7].

**Fig. 1:**
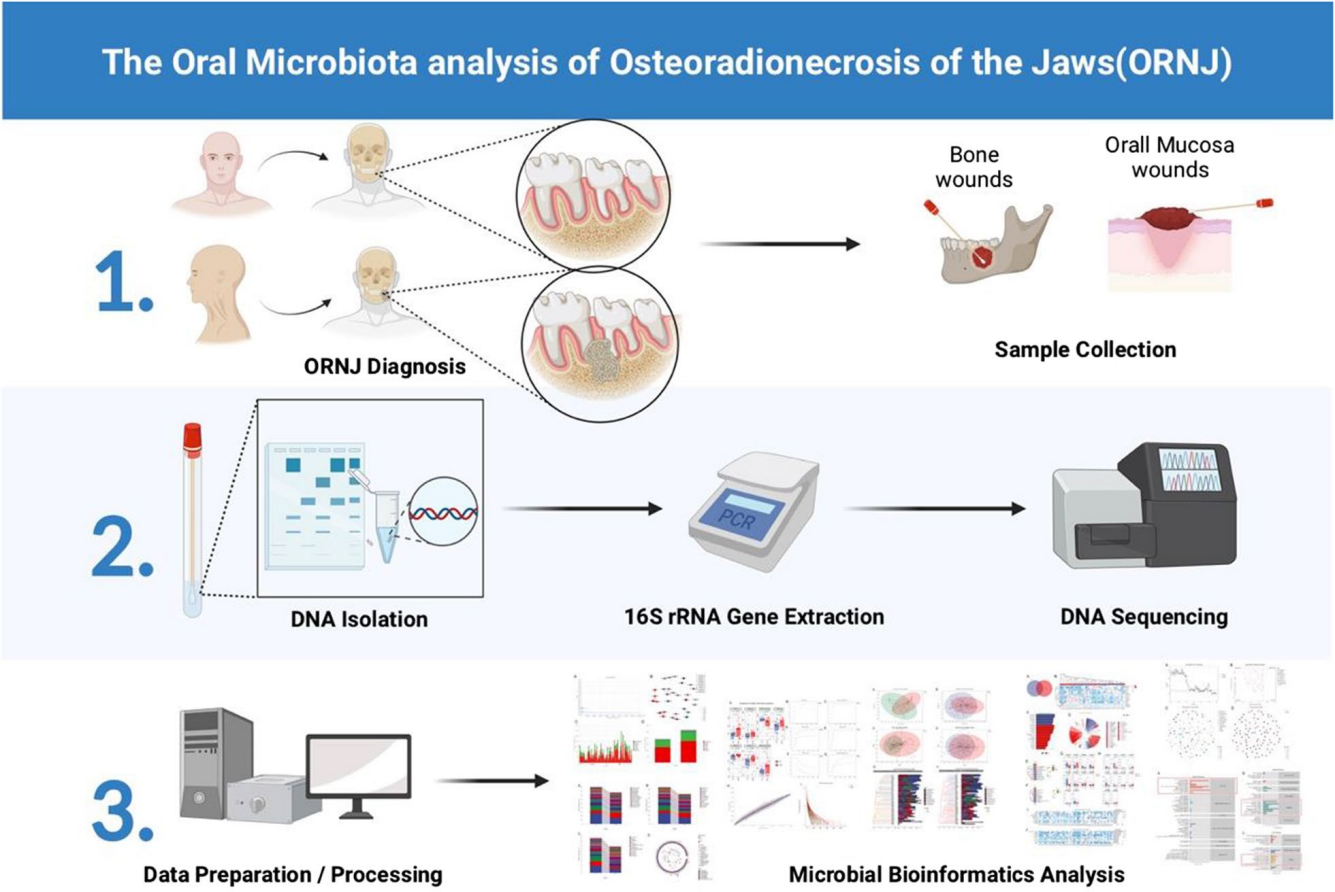
Role of oral microorganisms in radiation-induced ORN. Oral swabs were collected from the ORN lesions and contralateral normal tissues, and the abundance of different microbial specific was analyzed by high-throughput sequencing and bioinformatics

Radiotherapy also causes significant damage to the salivary glands and oral mucous membranes of HNC patients. The destruction of salivary glands leads to a significant decrease in saliva production, which along with a damaged mucosal epithelium creates ideal conditions for bacterial growth and colonization in the oral cavity [8]. Recent studies suggest that the oral microbiota plays a key role in the pathogenesis of ORN [9]. For instance, the abundance of *Actinomycetes* is increased in the oral cavity of patients with ORN [10], and *Streptococcus intermedius* also predominates the oral microflora in irradiated patients [11]. Unlike other types of osteomyelitis, ORN shows chronic development and lacks a clear boundary despite forming a sequestrum. Osteomyelitis is routinely treated with sequestrectomy or cortical osteotomy combined with antimicrobial therapy. However, the guidelines for monotherapy or combination therapy for ORN are yet to be defined [12]. In many cases, broad-spectrum antibiotics are the only option due to the limitations of current microbial identification techniques, and may lead to bacterial resistance and unsatisfactory treatment outcomes. Therefore, it is essential to identify the bacterial communities associated with ORN in order to treat the infection promptly and reduce mortality rates. However, most studies have only identified individual species in the oral microflora of ORN patients, and there are no definitive reports on the association between microbial changes and disease progression. We manage to investigate the role of oral microbiome in the etiology of ORN from the perspective of oral microecology in order to elucidate the relationship between the two more comprehensively and provide new guidance for the prevention and treatment of the disease. This is also the minority that explored the microbial composition of ORN using culture-independent methods, and there have been few previous studies of exposed bone tissue exudates to identify pathogenic bacteria (Fig. 1).

Traditional single bacterial culture and identification methods are still the gold standard for establishing the presence or absence of specific bacteria. However, these techniques are time-consuming and less sensitive, and cannot be applied to the analysis of a microbiome. On the other hand, high-throughput sequencing technology can rapidly and comprehensively map the microbiome of any tissue, and determine its function in diseases. Currently, 16s rRNA sequencing is the most widely used high-throughput approach for bacterial identification since it can generate a large amount of genomic information and calculate the relative abundance of almost all microbial species [13].

In this study, we analyzed the microbiota of radiation-induced ORN lesions and contralateral normal tissues isolated from the oral cavity of HNC patients using 16 S rRNA sequencing. We used normal tissues from the patients rather than healthy subjects as the control arm in order to negate the influence of genotype and diet on the oral flora. Our findings provide new insights into the role of the oral microbiota in the development of radiation-induced osteomyelitis, which can be useful for further mechanistic exploration and identification of diagnostic and predictive biomarkers of ORN.

## Materials and methods

### Study population

A total of 30 patients with ORN who presented to the Department of Oral Maxillofacial-Head and Neck Oncology, Shanghai Ninth People’s Hospital, Shanghai Jiao Tong University School of Medicine between January 2021 and December 2022 were included in this study. All patients manifested the typical clinical symptoms of ORN, such as bone exposure, osteonecrosis, fistula formation, inflammatory infiltration and soft tissue ulceration, after receiving high-dose radiation for primary neck tumor. Bone destruction was confirmed by imaging tests, which led to the diagnosis of ORN.

The exclusion criteria were as follows: (1) use of antibiotics or antibacterial drugs within the month preceding the study, (2) other infectious diseases, (3) diabetes, autoimmune diseases, and other systemic diseases, (4) other situations that contraindicated participation in the study. All subjects volunteered to participate in this study and provided written informed consent. This study was approved by the Ethics Committee of the Shanghai Ninth People’s Hospital Affiliated to Shanghai Jiao Tong University School of Medicine.

### Sample collection

All patients underwent segmental resection or debridement of sequestrum using intraoral or extraoral methods under general anesthesia, and some patients underwent flap transfer repair simultaneously. These patients required debridement to control osteomyelitis after removal of surface necrotic tissue in order to avoid contamination of soft tissue or sinus tracts. Swab samples were collected from the affected side and the contralateral healthy tissues. The patients were instructed to not eat, smoke or drink starting 2 h before sampling. After washing the wounds with sterile saline and hydrogen peroxide, the patients were instructed to rinse their mouth with water for 10  s and spit out the effluent. The mucosa or bone surface of the lesion (exposed) and the contralateral healthy tissue were swabbed separately, and the samples were collected into tubes. The swab samples were transported on ice and stored at − 80 °C. One part was used for post-stock routine microbial culture and the other for 16 S rRNA sequencing. Since one patient had low microbial abundance on the unaffected side, only 59 samples were included in the final analysis.

### DNA extraction

Total genomic DNA was extracted using the OMEGA Soil DNA Kit (M5635-02) (Omega Bio-Tek, Norcross, GA, USA) according to the manufacturer’s instructions, and stored at − 20 °C. The quantity and quality of the DNA samples were evaluated using the NanoDrop NC2000 spectrophotometer (Thermo Fisher Scientific, Waltham, MA, USA) and agarose gel electrophoresis respectively.

### PCR amplification and 16 S rRNA gene sequencing

The bacterial 16S rRNA gene V3-V4 region was amplified using the following primers: forward 338F 5′-ACTCCTACGGGAGGCAGCA-3′ and reverse 806R 5′-GGACTACHVGGGTWTCTAAT-3′. The reference database was HOMD (https://www.homd.org/). The PCR reaction mix consisted of 5 µL buffer (5×), 0.25 µL Fast Pfu DNA Polymerase (5 U/µL), 2 µL (2.5 mM) dNTPs, 1 µL (10 µM) each of forward and reverse primer, 1 µL DNA template and 14.75 µL ddH2O. The cycling parameters were as follows: initial denaturation at 98 °C for 5 min, followed by 25 cycles consisting of denaturation at 98 °C for 30 s, annealing at 53 °C for 30  s, and extension at 72 °C for 45 s, with a final extension of 5 min at 72 °C. PCR amplicons were purified using Vazyme VAHTSTM DNA Clean Beads (Vazyme, Nanjing, China) and quantified using the Quant-iT PicoGreen dsDNA Assay Kit (Invitrogen, Carlsbad, CA, USA). After the individual quantification step, amplicons were pooled in equal amounts, and pair-end 2*250 bp sequencing was performed using the Illumina NovaSeq platform with NovaSeq 6000 SP Reagent Kit (500 cycles) at Shanghai Personal Biotechnology Co. Ltd (Shanghai, China).

### Sequence analysis

Raw data were stored in FASTQ format (R1.fastq and R2.fastq, and Read 1 and Read 2 sequences were paired one by one). DADA2 was used for sequence denoising or clustering. Sample-specific 7 bp barcodes were incorporated into the primers for multiplex sequencing. Microbiome bioinformatics were performed using QIIME2 2019.4 [14]  with slight modifications (https://docs.qiime2.org/2019.4/tutorials/). Briefly, raw sequence data were demultiplexed using the demux plugin followed by primers cutting with cutadapt plugin [15]. Sequences were then quality filtered, denoised and merged, and chimera was removed using the DADA2 plugin [16]. Non-singleton amplicon sequence variants (ASVs) were aligned with mafft [17] and used to construct a phylogeny with fasttree2 [18]. Alpha-diversity metrics, Observed species, Shannon, Simpson, Faith’s PD Pielou’s evenness, Good’s coverage, beta diversity metrics (weighted UniFrac [19] and unweighted UniFrac [20], Jaccard distance, and Bray-Curtis dissimilarity) were estimated using the diversity plugin with samples rarefied to 47,983 sequences per sample. Taxonomy was assigned to ASVs using the classify-sklearn naïve Bayes taxonomy classifier in feature-classifier plugin [21] against the HOMD (https://www.homd.org/) database [22].

### Bioinformatics analysis

Sequence data analyses were performed using QIIME2. ASV-level alpha diversity indices, such as Chao1 richness estimator, Observed species, Shannon diversity index, Simpson index, Faith’s PD, Pielou’s evenness and Good’s coverage were calculated using the ASV table in QIIME2, and visualized as box plots. ASV-level ranked abundance curves were generated to compare the richness and evenness of ASVs among samples. Beta diversity analysis was performed to investigate the structural variation of microbial communities across samples using Jaccard metrics, Bray-Curtis metrics and UniFrac distance metrics, and visualized via principal coordinate analysis (PCoA), nonmetric multidimensional scaling (NMDS) and unweighted pair-group method with arithmetic means (UPGMA) hierarchical clustering [23]. Venn diagram was generated to visualize the shared and unique ASVs among samples or groups regardless of their relative abundance using R package “VennDiagram” [24].

Principal component analysis (PCA) was also conducted based on the genus-level compositional profiles. The taxonomy compositions and abundance were visualized using MEGAN [25] and GraPhlAn [26]. Taxa abundance at the ASV levels were compared among samples or groups by MetagenomeSeq, and visualized as Manhattan plots [27].

LEfSe (linear discriminant analysis effect size) was performed to detect the differentially abundant taxa across groups using the default parameters [28]. OPLS-DA (orthogonal partial least squares discriminant analysis) was also introduced as a supervised model to identify the microbiota variation among groups using the R package “muma” [29].

Random forest analysis was applied to discriminate the samples from different groups using QIIME2 with default settings. Nested stratified k-fold cross validation was used for automated hyperparameter optimization and sample prediction. The number of k-fold cross-validations was set to 10.

Co-occurrence network analysis was performed using SparCC and the pseudo count value was set to 10–6. The cutoff of correlation coefficients was determined as 70 through random matrix theory-based methods in the R package RMThreshold. Based on the correlation coefficients, we constructed co-occurrence network with nodes representing ASVs and edges representing correlations between these ASVs.

The network was visualized using R package “igraph” and “ggraph”. Microbial functions were predicted by PICRUSt2 (phylogenetic investigation of communities by reconstruction of unobserved states) based on the MetaCyc (https://metacyc.org/) and KEGG (https://www.kegg.jp/) databases.

### Statistical analysis

Sequence data analyses, box plots of alpha/beta diversity indice, manhattan plots, and microbiota variation plots were generated using the packages in the R package (v4.0.2, https://www.r-project.org/). Significant differences in alpha diversity between groups were verified by Kruskal-Wallis rank sum test. Wilcoxon test dunn’test used as post-hoc test. The significance of the differences in microbiota structure between the two groups was assessed by PERMANOVA (Permutational multivariate analysis of variance), ANOSIM (Analysis of similarities) [30] and Permdisp [31]. P < 0.05 was considered statistically significant.

## Results

### Characteristics of study participants and sequencing analysis

There were 16 males and 14 females (1.14:1) in our cohort, with median age of 57.5 (53.25, 63) years. Most patients (86.7%) had mandibular lesions. In addition, HNSCC was the predominant primary tumor (90%), and most patients had undergone tumor resection (90%). Some patients had experienced postoperative adverse reactions, such as infection, bleeding, and limited mouth opening. Two patients underwent tooth extraction after surgery. The clinical information of the patients is summarized in Table 1.

**Table 1:** Clinical characteristics of ORN cases

No.	Gender	Age	Position	Primary tumor	Tumorectomy	Supplement
**1**	Female	57	Right mandibular	SCC (tongue)	Yes	
**2**	Male	57	Left maxilla	SCC (gingival)	Yes	
**3**	Female	65	Left maxilla	SCC (gingival)	Yes	
**4**	Male	59	Right mandibular	SCC (gingival)	Yes	Postoperative recurrent infection with bleeding
**5**	Female	59	Right mandibular	SCC (floor of the mouth)	Yes	Postoperative recurrent infection
**6**	Female	75	Left mandibular	SCC (tongue)	Yes	Postoperative tooth extraction
**7**	Male	68	Left mandibular	SCC (gingival)	Yes	Postoperative limitation of mouth opening
**8**	Male	68	Left mandibular	Carcinoma of tonsil	Yes	
**9**	Female	64	Left mandibular	SCC (gingival)	Yes	Postoperative tooth extraction with labial numbness
**10**	Male	63	Bilateral mandibular	NPC	No	
**11**	Female	63	Right mandibular	SCC (tongue)	No	Postoperative recurrent infection
**12**	Male	62	Left mandibular	SCC (tongue)	Yes	
**13**	Female	62	Right mandibular	SCC (floor of the mouth)	Yes	
**14**	Male	59	Left mandibular	SCC (oropharynx)	Yes	
**15**	Male	58	Right mandibular	Neuroendocrine tumor	No	Postoperative limitation of mouth opening
**16**	Male	57	Left mandibular	SCC (tongue)	Yes	
**17**	Male	56	Left mandibular	SCC (buccal)	Yes	Postoperative recurrent infection with fistulae
**18**	Male	56	Left mandibular	SCC (buccal)	Yes	
**19**	Female	55	Left mandibular	SCC (gingival)	Yes	Postoperative recurrent infection with fistulae
**20**	Female	54	Left mandibular	SCC (tongue)	Yes	
**21**	Male	53	Left mandibular	SCC (floor of the mouth)	Yes	Postoperative recurrent infection
**22**	Male	49	Right mandibular	SCC (gingival)	Yes	Postoperative recurrent infection
**23**	Male	49	Bilateral mandibular	SCC (floor of the mouth)	Yes	
**24**	Female	48	Left mandibular	SCC (tongue)	Yes	
**25**	Female	48	Left mandibular	SCC (tongue)	Yes	
**26**	Female	47	Right mandibular	SCC (tongue)	Yes	Postoperative recurrent infection with fistulae
**27**	Female	46	Left mandibular	SCC (gingival)	Yes	
**28**	Female	65	Right maxilla	SCC (buccal)	Yes	Postoperative recurrent infection
**29**	Male	51	Left maxilla	SCC (buccal)	Yes	
**30**	Male	75	Left mandibular	SCC (floor of the mouth)	Yes	

**Fig. 2:**
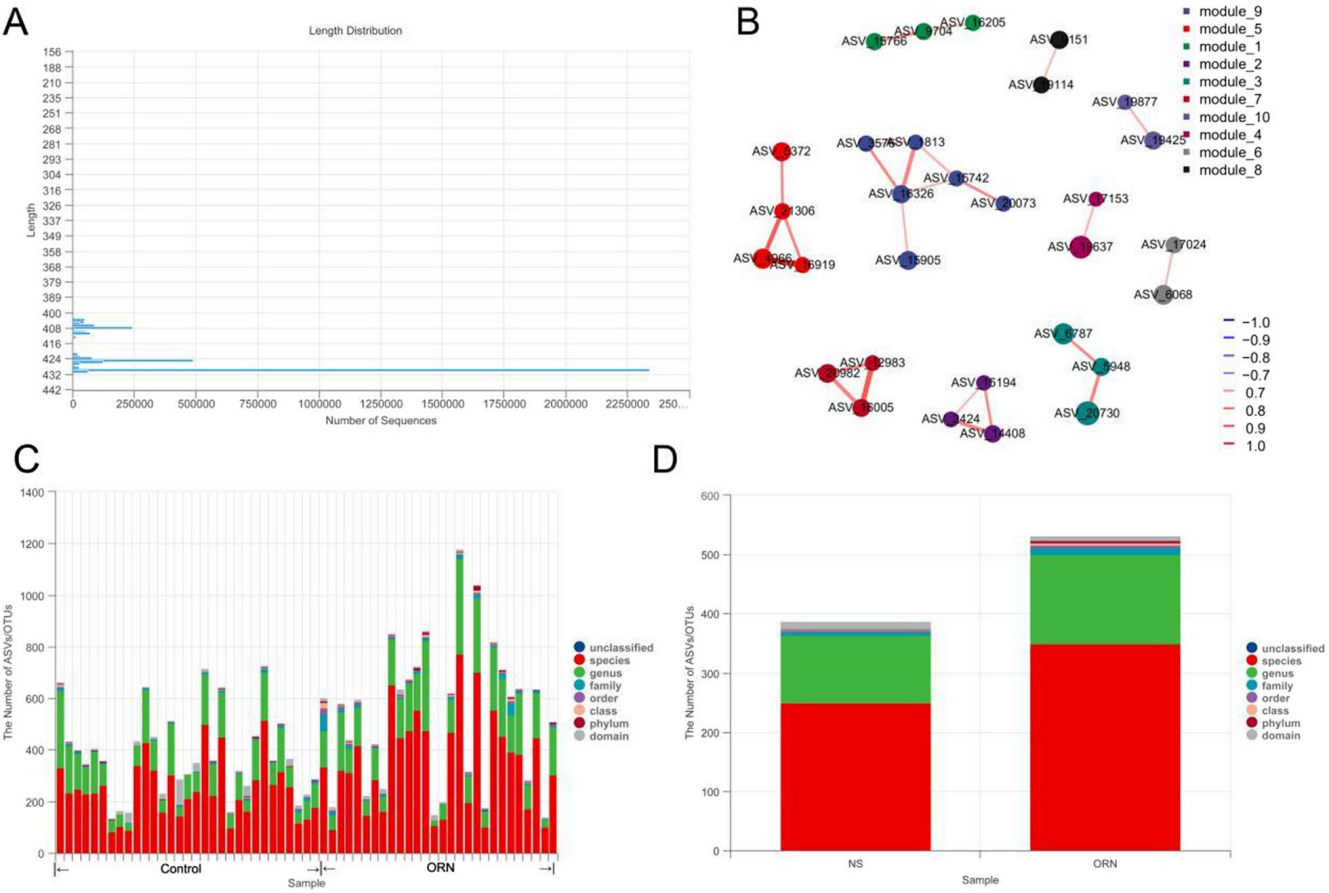
General characteristics of sample sequence. **A** Sequence length distribution. **B** ASV results. **C–D**. Differences in the microbiota of ORN and control groups at each level

A total of 4583440 initial reads were obtained after 16 rRNA sequencing. Following primer removal, quality filtering, denoise, splicing and chimerism removal, 3821418 high-quality sequences were obtained, and their lengths ranged from 366 to 432 bp (Fig. 2A). The results of ASV flattening also verified the fidelity of the results (Fig. 2B). Therefore, the reads obtained in the samples were considered sufficient for species taxonomic annotation and composition. Overall, 15 phyla, 36 classes, 65 orders, 123 families, 241 genera and 668 species of oral microorganisms were obtained, and significant differences were observed between the ORN and control groups at all levels (Fig. 2C, D).

### Microbiota composition analysis between ORN and NS Group

After removing singleton sequences, we observed significant differences between the microbial composition of the two groups at three taxonomic levels: family, genus, and species. We screened the top 15 oral microorganisms that were significantly different and highly abundant.

**Fig. 3:**
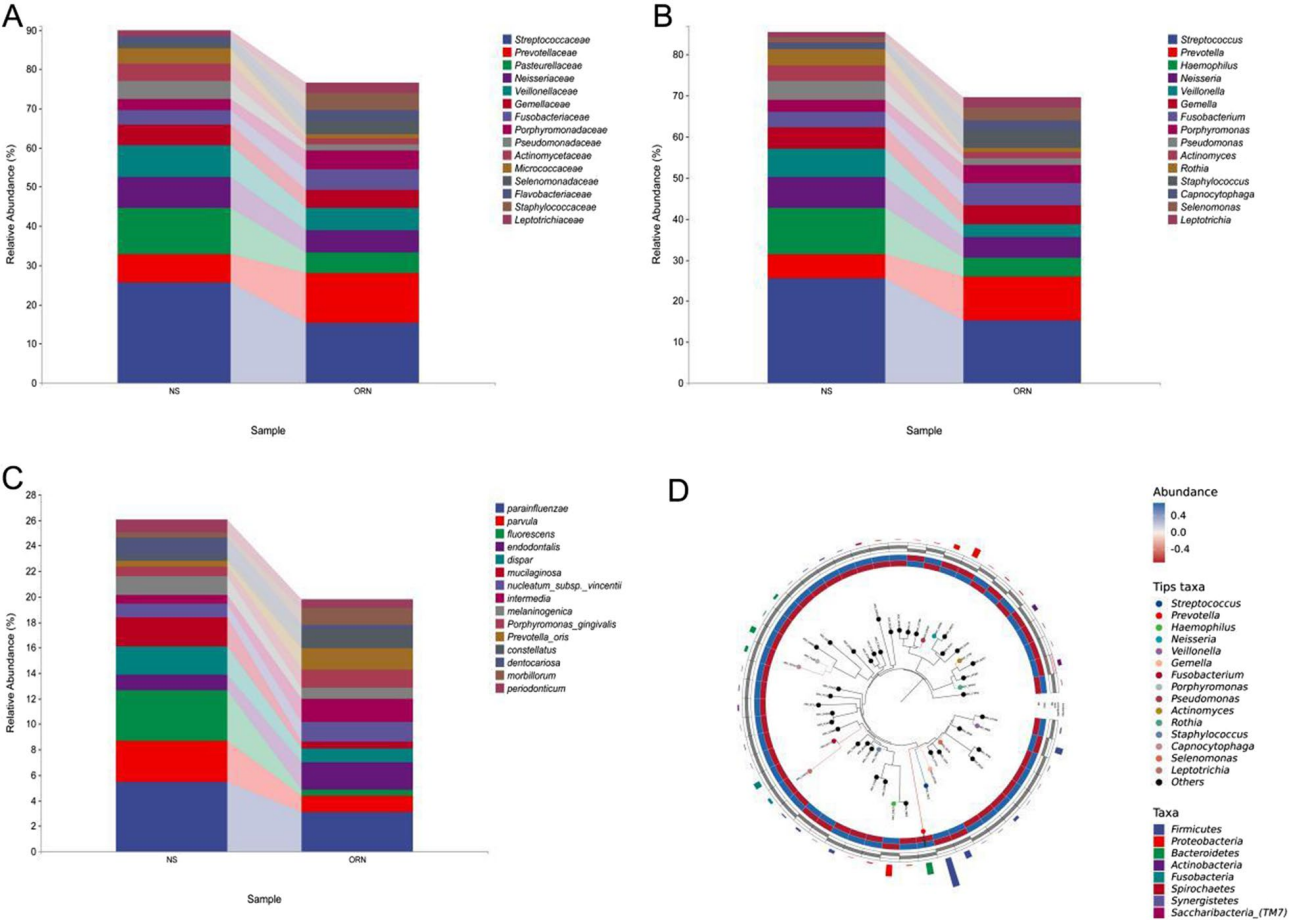
The oral microbiota profiles of ORN and control group. **A–C**. The significant differences and relative-high abundances between ORN and NS Group (statistically significant between-group comparisons, p < 0.05) **D**. Ggtree of the microbial composition and abundance in each group

At the family level, the relative abundance of Prevotellaceae was significantly increased in the ORN group. In addition, Fusobacteriaceae, Porphyromonadaceae, Actinomycetaceae and Staphylococcaceae also showed increased abundance. In contrast, the relative abundance of Streptococcaceae, Pasteurellaceae and Micrococcaceae was markedly lower in the ORN group compared to that in the control group (Fig. 3A). Furthermore, *Prevotella* and *Staphylococcus* were the abundant genera in the ORN group, whereas the control group showed higher abundance of *Streptococcus*, *Haemophilus*, *Neisseria* and *Veillnoella* (Fig. 3B). Although it is reasonable to surmise that the abundant microbes in the control group may protect against ORN, *Neisseria* is often considered as the causative agent of ORN. At the species level, the ORN group had higher abundance of *endodontalis* and *intermedia*, while that of *parvula*, *fluorescens*, and *mucilaginosa* was extremely low (Fig. 3C). Therefore, these abundant species likely play a key role in the development and progression of ORN. We constructed an evolutionary tree using ggtree and as shown in Fig. 3D, *Prevotella* and *Streptococcus* were at critical taxonomic positions in the ORN group (Fig. 3D).

### Alpha diversity analysis of the oral microbiota

**Fig. 4:**
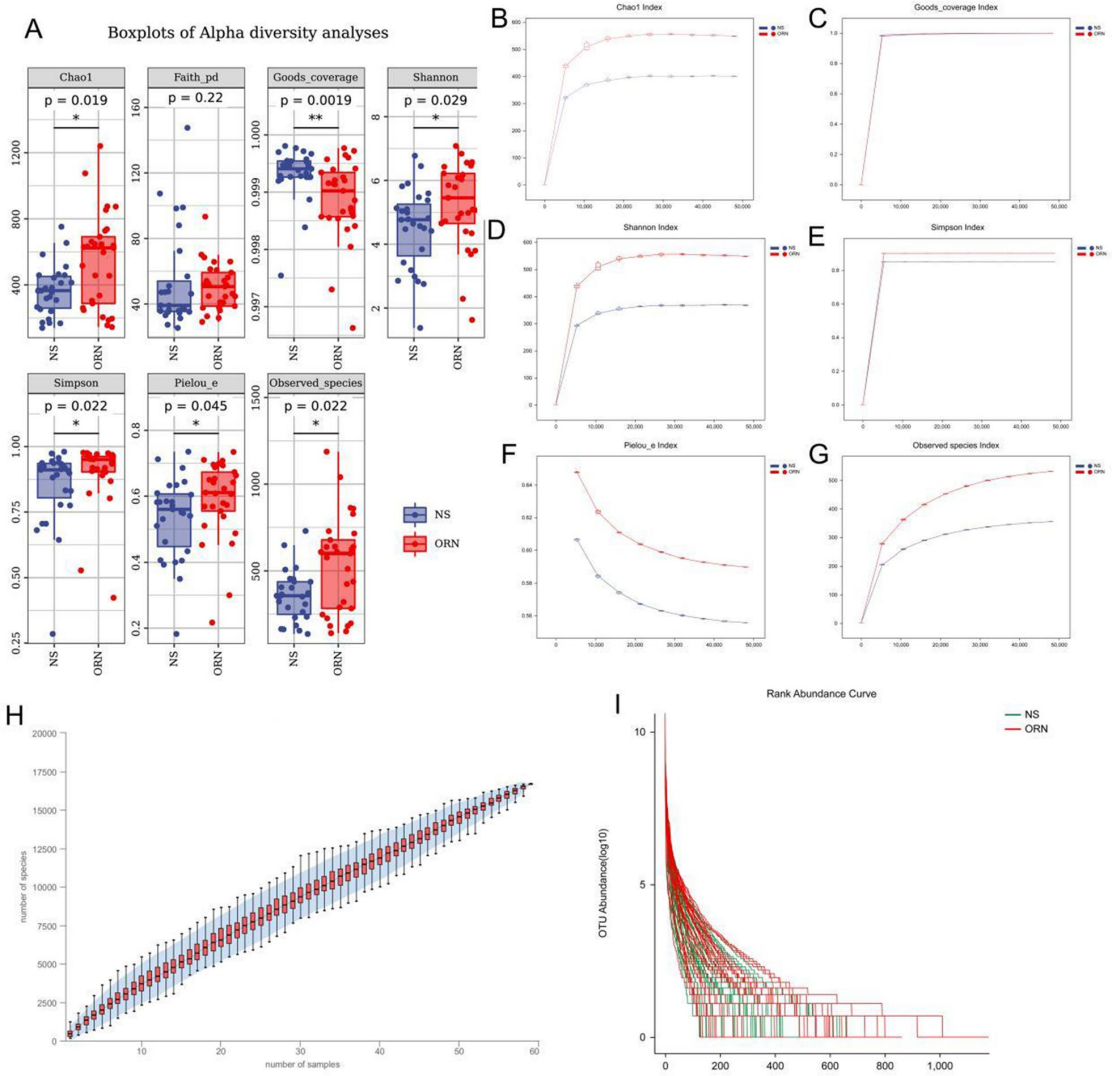
Differential microbial diversity between ORN and control groups. **A**. Boxplots of Alpha diversity analyses. **B–G**. Different evaluation indices for diversity. **H**. Species accumulation curves **I**. Rank abundance curve for ORN and control groups

The alpha diversity of the oral microbiota in the two groups was also compared (Fig. 4A). The Chao1 (p = 0.019, Fig. 4B) and Observed species (p = 0.022, Fig. 4G) indices differed significantly, which corresponded to greater species richness and abundance in the ORN. The Shannon (p = 0.029, Fig. 4D) and Simpson (p = 0.022, Fig. 4E) indices were also significantly different, with greater species diversity in the ORN group compared to the controls. In addition, differences in the Pielou ‘s evenness index suggested a more uniform distribution of species in the ORN group (p = 0.045, Fig. 4F). On the other hand, the Good’s coverage index was significantly lower in the ORN group compared to the controls, indicating less species coverage in the former (p = 0.0019, Fig. 4C). However, the Faith’s PD index did not differ significantly between the two groups, suggesting that evolutionary-based diversity was similar and ORN did not increase microbial variation. Species accumulation curves further indicated that the current sample size of both groups was sufficient to reflect the species composition of the oral microbial community (Fig. 4H). According to the abundance rank curves of the highly abundant and aggregated ASVs (Fig. 4I), the oral microbiota in the ORN group had greater alpha diversity.

### Beta diversity analysis of the association between microbial diversity and ORN

**Fig. 5:**
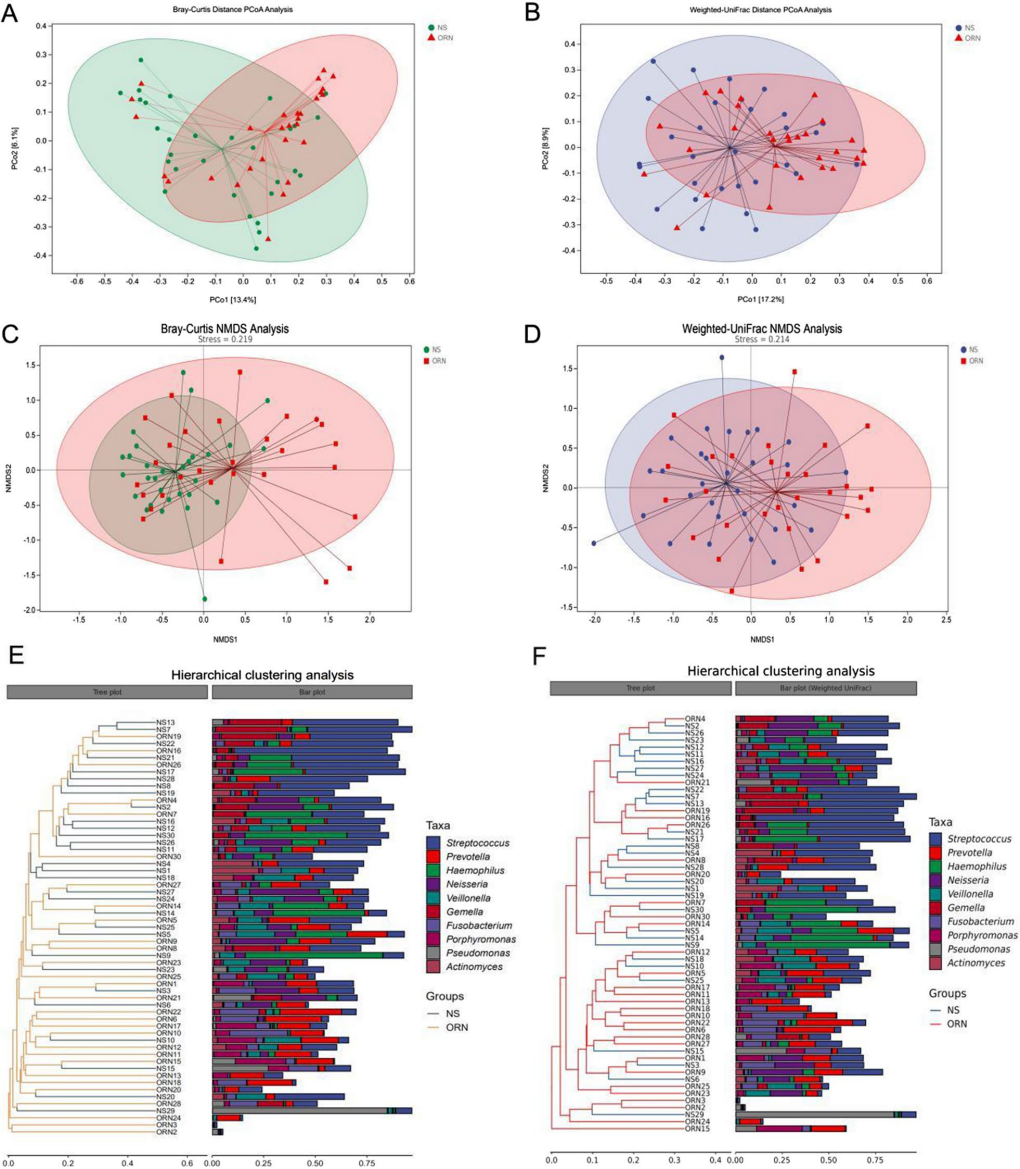
Differential microbial diversity in the ORN and control groups. **A–D**. PCA plots of ORN and control groups based on oral microbiota analyses (one plot represents one sample, and the large ellipses in each group represent the 95% confidence interval (CI) range of the matching group. Paired samples were selected for this group). **E**, **F**. Hierarchical clustering analysis showed similarity between samples

Since Jaccard distance and unweighted UniFrac distance algorithms perform poorly regarding species abundance, we further examined beta diversity between the two groups using the Bray-Curtis distance and weighted UniFrac distance. Principal coordinates analysis (PCoA) showed significant differences between the two groups (Fig. 5A, B), and the microbiota was more concentrated in ORN lesions. In addition, the oral microbiota composition of the ORN samples was more similar. This further confirmed that radiation therapy can alter the oral microbial composition. Non-metric multidimensional scaling analysis (NMDS) after one-step dimension reduction showed that despite the small distance between the ORN samples, the change in the species was more obvious within the ORN group and the gap was large (Fig. 5C, D). Hierarchical clustering further demonstrated similarity between samples, suggesting similar patterns of radiation-induced microbial changes in the oral cavity during progression to ORN (Fig. 5E, F). *Prevotella* was more concentrated in ORN, while *Streptococcus* and *Haemophilus* were more densely distributed in the control group. And, briefly, trending alterations in microbial diversity and community composition are rapidly achieved in changes in the oral environment (receiving irradiation).

### Species difference and marker species analysis

**Fig. 6:**
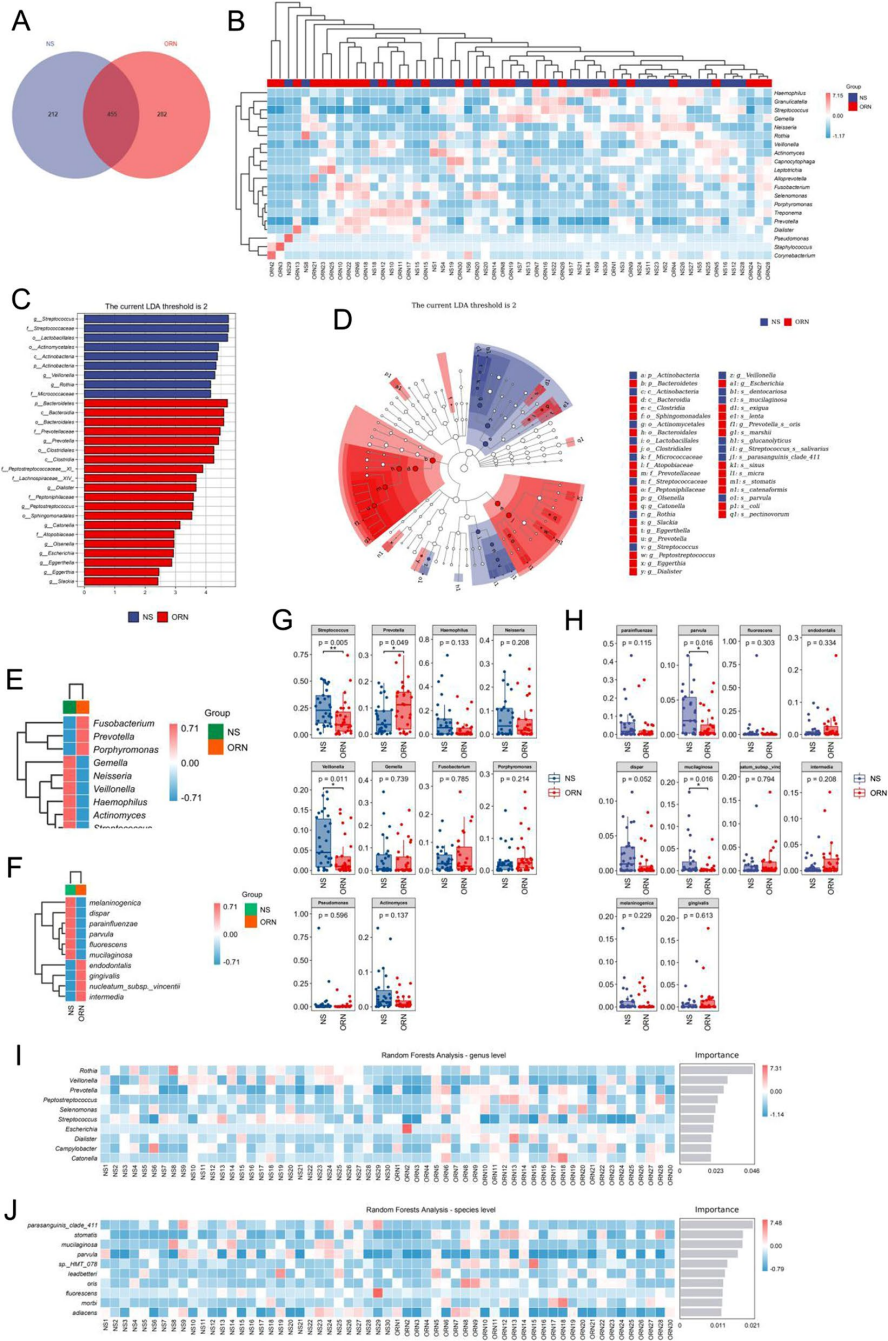
Species differences between the ORN and control groups. **A** Community analysis Wayne plot of ASV in two groups. **B** Abundance and distribution trend of the top 20 genera. **C** LEfSe analysis of significant differences at the genus level. **D** Clodogram of taxonomic hierarchy. **E**,** F**. Top 10 genera and species in both groups. **G**,** H**. Microorganisms with significant differences at the genus and species level. **I**, **J**. Random Forest analysis plots of the significant microorganisms

According to community analysis, there were 737 characteristic ASVs in the ORN group, 667 in the control group, and 455 common to both groups (Fig. 6A). We further compared the species composition between samples and analyzed the distribution of the top 20 abundant genera. As shown in Fig. 6B, *Prevotella* had significantly higher abundance in the ORN group compared to that in the control group, whereas *Streptococcus* showed the opposite trend. LefSe analysis showed that *Streptococcus*, *Veillonella* and *Rothia* were the significantly different species in the control group, while *Prevotella* and *Dialister* were significantly different in ORN (Fig. 6C). The cladogram of the hierarchical taxonomic distribution of marker species in each group is shown in Fig. 6D, and the top 10 genera and species in each group are shown in the heat maps in Fig. 6E, F. *Streptococcus* and *Prevotella* were the significantly different genera, and *parvula* and *mucilaginosa* species showed significant differences (Fig. 6G, H). Random forest analysis further validated the relevance of g_*Prevotella*, g_*Streptococcus*, s_*parvula* and s_*mucilaginosa* as microbial markers (Fig. 6I, J).

### Association network analysis

**Fig. 7:**
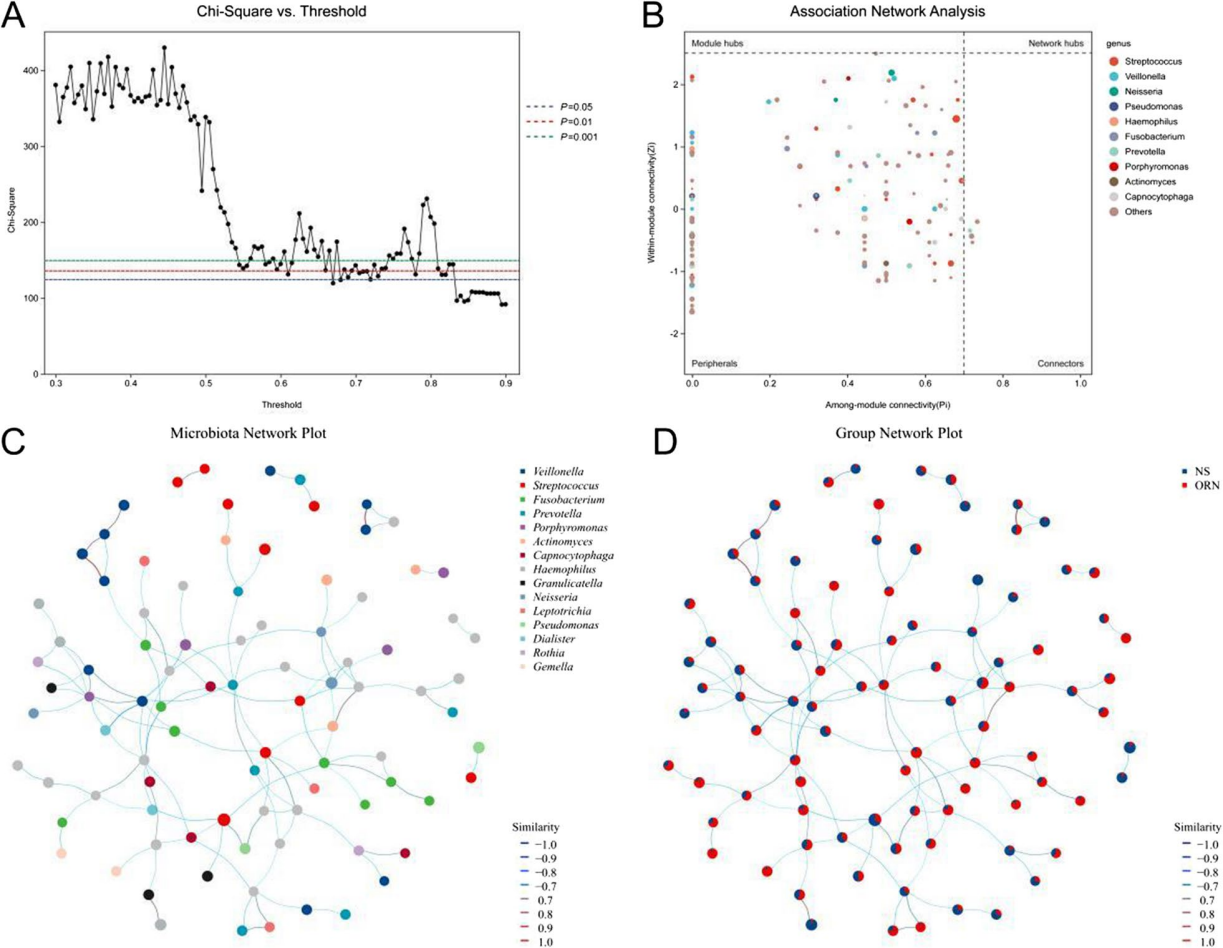
The oral microbiota profiles of ORN and control groups at the three levels. **A** Association network showing the composition of the microbiota. **B** Association network showing major microorganisms. **C** Microbiota network plot showing a co-occurrence trend in ORN. **D** Group network plot showing a co-exclusion trend in control group

We used the SparCC method to estimate association networks of the microbial communities. As shown in Fig. 7A, there was significant association between the majority of the microbial species in ORN. Furthermore, g_*Streptococcus*, g_*Veillonella*, g_*Neisseria*, g_*Pseudomonas*, g_*Haemophilus*, g_*Fusobacterium*, and g_*Prevotella* were the key microorganisms in the ecological network (Fig. 7B). Further mapping of the association network indicated that g_*Streptococcus* and specific microbial communities presented an inherent pattern of co-occurrence driven by spatiotemporal changes and environmental processes in the ORN group (Fig. 7C). On the other hand, the control group showed a negative correlation pattern of co-exclusion (Fig. 7D). These results were indicative of a global imbalance in species diversity and ecological diversity in the oral microecology of ORN.

### Metabolic pathways associated with the abundant microorganisms in ORN

**Fig. 8:**
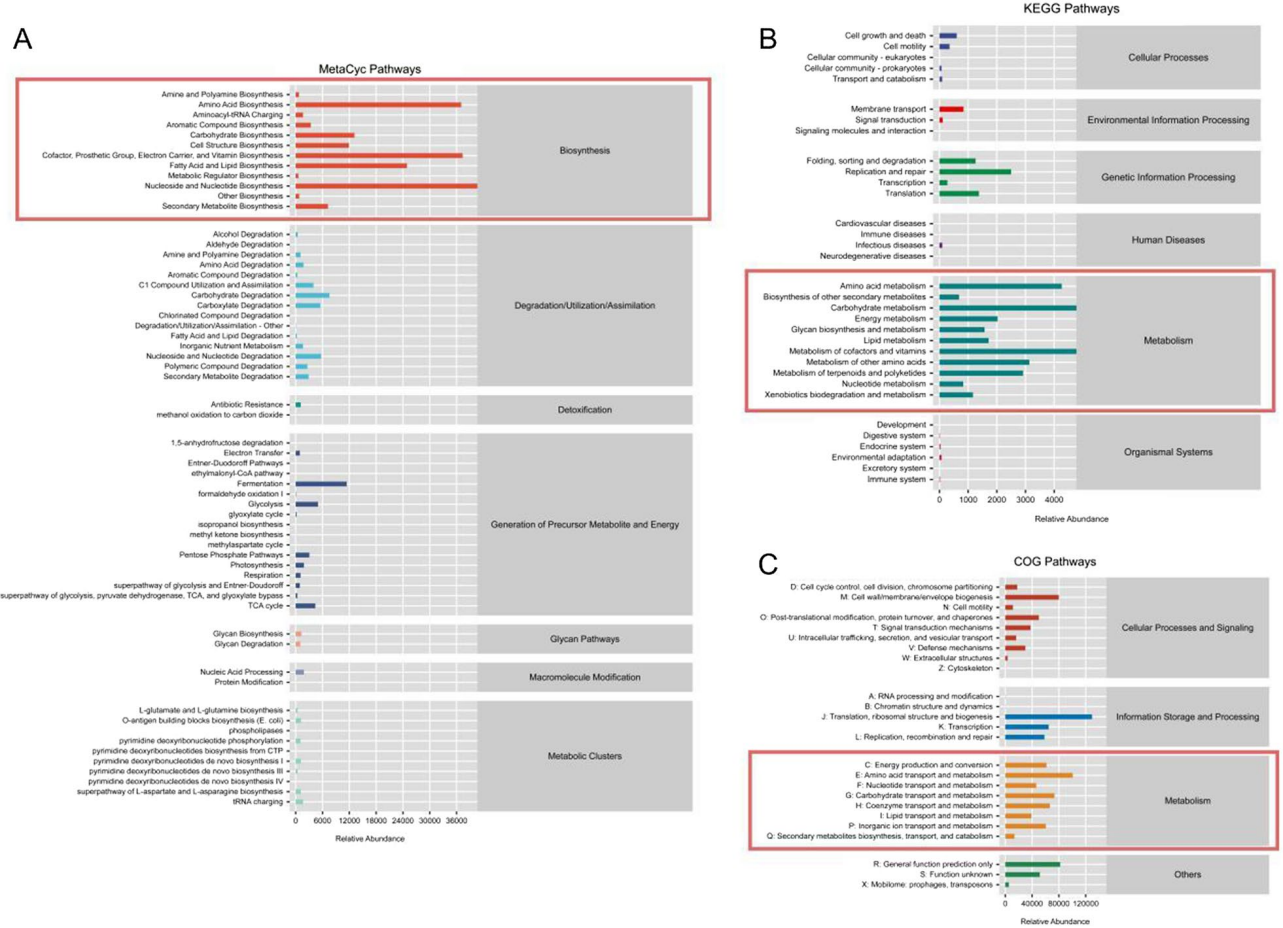
Metabolic pathways associated with the abundant microorganisms in ORN. **A** MetaCyc Pathways. **B** KEGG pathways. **C** COG pathways

We next identified the metabolic pathways associated with the abundant microorganisms in ORN. MetaCyc analysis showed enrichment of pathways related to the biosynthesis of nucleosides and nucleotides, vitamins, amino acids, fatty acids and lipids, cofactor, prosthetic group and electron carriers. The significantly enriched KEGG pathways included those related to the metabolism of carbohydrates, cofactors and vitamins, and amino acids, as well as pathways involved in the processing of genetic information. Furthermore, COG analysis also showed that the abundant microbial species in ORN were associated with the transport and metabolism of amino acids, carbohydrates and coenzymes, along with translation, ribosomal structure and biogenesis in the information storage and processing. Taken together, the dominant microbiota in the ORN lesions may disrupt bone regeneration by enhancing osteoclast activity through these metabolic pathways (Fig. 8).

## Discussion

Osteoradionecrosis (ORN) is one of the serious complications of radiation therapy for head and neck cancer, and is common in middle-aged men. It primarily affects the mandible due to significant reduction in blood supply and greater load-bearing compared to the maxilla [32]. In addition, due to differences in the bone density, the mandible absorbs a higher amount of radiation compared to the maxilla [33]. Clinically, ORN ranges from asymptomatic cases with intact mucosa to severe cases with exposed bone surfaces and severe infection [34]. Contrary to the long-held consensus that ORN is an aseptic necrotic condition, the bacteria in the oral cavity may play a key role in the development of ORN, especially since it is one of the microbe-enriched sites in the human body with more than 700 microbial species [35, 36]. The role of oral microbiome in the development of ORN cannot be ignored. Radiation damages the blood vessels and bone cells, causing mucosal or skin ulceration that exposes the alveolar process and jaw bone surface, leaving unhealed wounds that are highly susceptible to anaerobic infections. In fact, the oral cavity is enriched in anaerobic bacteria, which may be responsible for chronic, refractory inflammation and numerous purulent secretions [37]. In addition, irradiated bones and soft tissues may lose their natural resistance, resulting in microbial invasion [38], which leads to inflammation and necrosis of soft tissues and bone tissues through virulence factors. The ORN samples used in this study were taken from tissues with infection, fistulae, pus discharge and exposed bone surfaces, and none of the patients showed reparative bone tissue. The contralateral healthy tissues of the same patients were taken as controls to negate any effects of genotype and diet on the oral microbiota. We did not select patients without post-radiotherapy ORN since disturbing the bone tissue in such cases may induce ORN. The ORN group showed greater microbial abundance and more significant change in species diversity compared to that in the control group. The ORN lesions showed an increase in the relative abundance of f_Prevotellaceae, f_Fusobacteriaceae, f_Porphyromonadaceae, f_Actinomycetaceae, f_Staphylococcaceae, g_*Prevotella*, g_*Staphylococcus*, s_*Endodontalis* and s_*Intermedia*, while f_Streptococcaceae, g_*Streptococcus* and g_*Haemophilus* were more abundant in the normal tissues. Therefore, we hypothesized that g_*Prevotella* and g_*Streptococcus* may play functional roles in ORN lesions and healthy oral tissues respectively. Furthermore, g_*Prevotella*, g_*Streptococcus*, s_*Parvula* and s_*Mucilaginosa* were identified as potential diagnostic and prognostic biomarkers of ORN. Taken together, radiation-induced ORN is associated with changes in the oral microflora, which raises the possibility of using relevant bacteria as diagnostic markers for ORN.

*Prevotella* is a large genus of p_Bacteroidetes consisting of Gram-negative anaerobic bacteria that have been isolated from the oral cavity and gut of healthy individuals [39]. Studies show that the presence or relative increase in abundance of certain species of *Prevotella* is associated with inflammatory diseases and endogenous infections, such as rheumatoid arthritis, intestinal dysbiosis, bacterial vaginosis, asthma and periodontitis [40]. Among the periodontal microbial complexes, *P. intermedia*, *P. nigrescens* and *P. micros* belong to the orange complex in the pyramids of Socransky and Haffajee, are late colonizers of oral biofilms and play an important role in the progression of periodontitis [41]. *Prevotella* stimulates dendritic cells (DCs) to release IL-1β, IL-6 and IL-23 through toll-like receptor 2 (TLR2), which in turn mediates IL-17 production by T helper 17 (Th17) cells that activate neutrophils. Thus, *Prevotella* can promote periodontitis by driving neutrophil recruitment through Th17 immune responses, and chronic activation of the Th17 pathway may trigger the local bone loss and tissue destruction characteristic of periodontitis [42]. In addition, the local dysbiosis caused by radiation exposure transforms the normally commensal *Prevotella* to an opportunistic pathogen, which produces virulence factors such as adhesins, proteases, hemolysins, lipopolysaccharides (LPS) and extracellular polysaccharides [43]. LPS induced osteoclast formation and inhibited osteogenesis in the co-culture of bone marrow mononuclear cells and primary osteoblasts by stimulating secretion of transforming growth factor β (TGF-β) and prostaglandin E2 (PGE2) [44]. In addition, several biosynthesis and metabolism-related pathways were enriched among the abundant microorganisms in ORN, indicating that *Prevotella* may impair bone regeneration and lead to bone loss by enhancing osteoclast activity through these metabolic pathways, and its role in ORN is more complex than that of a mere contaminant. *Prevotella* is clearly associated with the clinical progression of ORN and is a potential prognostic biomarker.

The s_*Streptococcus* species includes Gram-positive bacteria, and most streptococci isolated from the oral cavity are alpha-hemolytic or non-hemolytic. Except for *S. mutans*, the main pathogen of caries, oral *Streptococcus* is a commensal genus and even includes beneficial microorganisms [45]. In addition, some oral streptococci can impede the formation of cariogenic streptococcal biofilms by acting on stimulatory peptides (CSPs) [46]. There are interactions between different bacteria in biofilms, and the symbiotic relationship between bacteria becomes more complex when the environment changes [47]. Streptococci were also abundant in the normal tissues, which suggests a potential protective role of these bacteria against ORN progression, although the related mechanisms still need further in-depth study. Goda et al. analyzed the core microbiome of chronic osteomyelitis of the jaw (COMJ), and found that *F. nucleatum* promotes biofilm formation and synergistically induces bone resorption with other pathogenic bacteria [48]. However, we did not observe a key involvement of *F. nucleatum*, in the pathogenesis of ORN. This can be attributed to the fact that COMJ includes primary COMJ (PCO), chronic suppurative osteomyelitis (SUP), ORN of the jaw and bisphosphonate-associated osteonecrosis of the jaw (BRONJ), and would therefore exhibit differences in microbiome composition.

There are some limitations in this study that ought to be considered. First, the oral swabs collected from the patients may have been contaminated with saliva, adjacent odontogenic lesions or blood-derived pathogens from other focal sources, which can potentially affect sequencing results. Secondly, 16 S rRNA sequencing cannot distinguish between expressed and unexpressed genes, and therefore has limited ability to provide information regarding the actual function of the microbiome. To overcome this limitation, and understand the exact role of these bacteria in ORN, it is necessary to combine metagenomic sequencing with transcriptomics and proteomics, or increase the sample size.

This study is the first to explore the microbial composition of ORN using culture-independent methods, and identify pathogenic bacteria in exposed bone tissue exudates. There were significant differences between the microbial composition of healthy and affected tissues in the oral cavity of HNC patients after radiotherapy, indicating a pivotal of the oral microbiota in post-radiation inflammatory response and osteonecrosis. Furthermore, *Prevotella* and certain other genera can serve as diagnostic and prognostic biomarkers of ORN, and may improve ORN management and treatment. Further studies with larger populations are needed to fully understand the specific mechanisms employed by the oral microbiota in the progression of ORN.

However, the limitation of this study is the limited number of individuals included. In terms of sampling, all operations are performed intraorally, and contamination with saliva, etc., may occur, while adjacent infection with blood from odontogenic lesions or other focal sources may also lead to changes in sample results. At the same time the ability of 16 S rRNA sequencing to provide information about the actual function of the microbiome is limited because this method cannot distinguish between expressed and unexpressed genes. We believe that in order to better elucidate the role of oral microbiome in ORN, it is necessary to increase the number of samples, as well as through metagenome and even transcriptome analysis, to provide better information on the microbiome and ORN correlation, so as to provide us with more disease management basis for the prevention and treatment of ORN.

## Conclusion

The oral microbiota, particularly *Prevotella*, plays a key role in the etiopathology of ORN. Furthermore, radiation exposure alters the abundance of pathogenic bacteria in the oral cavity in a dose-dependent manner. In addition to the direct impact of the pathogenic bacteria, the disruption caused in microbial communities and host immune responses can lead to aberrant cytokine secretion. Therefore, future studies must employ longitudinal sampling strategies, larger sample sizes, and deeper molecular and phenotypic characterization of patients and controls to better understand the etiology of ORN.

## Data Availability

The sequence data have been submitted to the NCBI Sequence Read Archive (Accession Number: PRJNA533177).
